# Is Treatment with Trimetazidine Beneficial in Patients with Chronic Heart Failure?

**DOI:** 10.1371/journal.pone.0094660

**Published:** 2014-05-05

**Authors:** Xiang Zhou, Jianchang Chen

**Affiliations:** Department of Cardiology, The Second Affiliated Hospital of Soochow University, Suzhou, Jiangsu, China; University of Louisville, United States of America

## Abstract

**Background:**

Whether additional benefit can be achieved with the use of trimetazidine (TMZ) in patients with chronic heart failure (CHF) remains controversial. We therefore performed a meta-analysis of randomized controlled trials (RCTs) to evaluate the effects of TMZ treatment in CHF patients.

**Methods:**

We searched PubMed, EMBASE, and Cochrane databases through October 2013 and included 19 RCTs involving 994 CHF patients who underwent TMZ or placebo treatment. Risk ratio (RR) and weighted mean differences (WMD) were calculated using fixed or random effects models.

**Results:**

TMZ therapy was associated with considerable improvement in left ventricular ejection fraction (WMD: 7.29%, 95% CI: 6.49 to 8.09, p<0.01) and New York Heart Association classification (WMD: −0.55, 95% CI: −0.81 to −0.28, p<0.01). Moreover, treatment with TMZ also resulted in significant decrease in left ventricular end-systolic volume (WMD: −17.09 ml, 95% CI: −20.15 to −14.04, p<0.01), left ventricular end-diastolic volume (WMD: −11.24 ml, 95% CI: −14.06 to −8.42, p<0.01), hospitalization for cardiac causes (RR: 0.43, 95% CI: 0.21 to 0.91, p = 0.03), B-type natriuretic peptide (BNP; WMD: −157.08 pg/ml, 95% CI: −176.55 to −137.62, p<0.01) and C-reactive protein (CRP; WMD: −1.86 mg/l, 95% CI: −2.81 to −0.90, p<0.01). However, there were no significant differences in exercise duration and all-cause mortality between patients treated with TMZ and placebo.

**Conclusions:**

TMZ treatment in CHF patients may improve clinical symptoms and cardiac function, reduce hospitalization for cardiac causes, and decrease serum levels of BNP and CRP.

## Introduction

Chronic heart failure (CHF) is a complex clinical syndrome characterized by decreased myocardial contractility, hemodynamic abnormality and neuroendocrine activation. There are multiple etiologies leading to this final common clinical pathway, which carries a 50% 5-year mortality rate and is responsible for over one third of all deaths in the United States from cardiovascular causes [1]. The past few decades have witnessed remarkable progress in the drug therapy for CHF. The clinical application of beta-adrenergic receptor blockers, angiotensin-converting enzyme inhibitors, angiotensin II receptor blockers and aldosterone receptor antagonists has significantly reduced cardiovascular events and mortality in patients with CHF [2]. However, CHF remains a leading cause of morbidity and mortality throughout the world.

Trimetazidine (TMZ), a piperazine derivative used as an anti-anginal agent, selectively inhibits mitochondrial long-chain 3-ketoacyl coenzyme A thiolase. By decreasing fatty acid oxidation and stimulating glucose utilization, TMZ restores coupling between glycolysis and carbohydrate oxidation, and leads to ATP production with less oxygen consumption. Previous studies have reported that TMZ exerts cardioprotective effects by reducing oxidative damage, inhibiting inflammation and apoptosis, and improving endothelial function [3]–[6].

Over the past few years, several small randomised controlled trials (RCTs) have been conducted to evaluate the effects of TMZ treatment in patients with CHF. These trials investigated clinical symptoms, cardiac function, quality of life, hospitalization, mortality and cardiovascular events, comparing TMZ with placebo. In addition, two meta-analyses of RCTs have also been performed to assess the therapeutic effects of TMZ in CHF patients [7], [8]. However, some conclusions drawn from these two meta-analyses are not consistent. We therefore performed an updated meta-analysis including a few recently published RCTs to provide more convincing evidence of TMZ therapy in patients with CHF.

## Methods

### Search strategy and selection criteria

We performed an electronic literature search of PubMed, EMBASE, and Cochrane databases through October 2013, using the terms “Trimetazidine”, “Vastarel”, “Idaptan”, “heart failure”, “cardiac failure”, “cardiac dysfunction”, “cardiac insufficiency”, “cardiomyopathy”, and “ventricular dysfunction”. Sensitive filters identified clinical trial or RCT in the Medline database and the EMBASE database. The search was limited to human subjects, with no restriction for language.

RCTs reporting at least one of the outcomes were considered eligible. These outcomes included cardiac function parameters (ie, left ventricular ejection fraction (LVEF), left ventricular end-systolic volume (LVESV), left ventricular end-diastolic volume (LVEDV)), New York Heart Association (NYHA) classification, exercise tolerance (ie, exercise duration), all-cause mortality, hospitalization, cardiovascular events, B-type natriuretic peptide (BNP), and C-reactive protein (CRP).

### Data extraction and quality assessment

Two investigators independently reviewed all potentially eligible studies using predefined eligibility criteria and collected data from the included trials. We extracted details on study characteristics, patient characteristics, inclusion criteria, ischemic etiology, intervention strategies, duration of follow-up, and clinical outcomes including LVEF, LVESV, LVEDV, NYHA classification, exercise duration, all-cause mortality, hospitalization, BNP and CRP. The quality of included RCTs was assessed by the Jadad scale [9], and a numerical score between 0 and 5 was assigned as a measure of study design.

### Statistical analysis

Dichotomous data were analyzed using risk ratio (RR) with 95% confidence intervals (CI), while continuous variables were analyzed using weighted mean differences (WMD) and 95% CI. The heterogeneity of results across trials was assessed using the Chi-square based Q-test. A p value>0.10 for the Q-test indicated a lack of heterogeneity among the studies. Thus, the pooled effect was calculated using fixed effects model. Otherwise, random effects model was applied in case of significant heterogeneity across studies. Sensitivity analysis was also conducted to assess the influence of each individual study on overall estimates by sequential removal of individual studies. All statistical analyses were performed using RevMan 5.0 (Cochrane Collaboration, Oxford, UK) and STATA software 10.0 (Stata Corporation, College Station, Texas, USA). P<0.05 was considered statistically significant.

## Results

### Eligible studies

**Figure 1:**
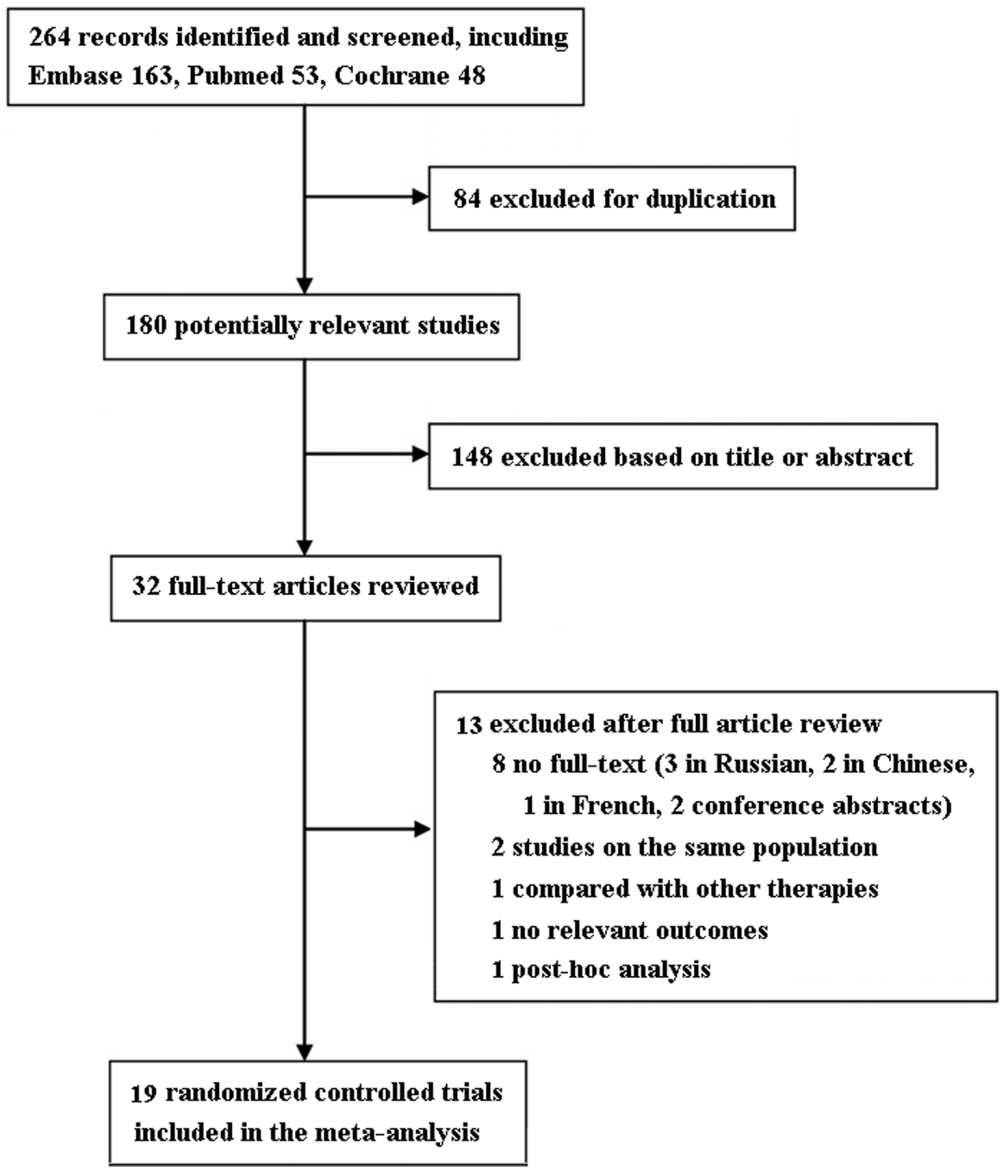
Flow diagram of eligible studies included in the meta-analysis.

The flow of selection of studies for the meta-analysis is shown in Figure 1. Among the initial 264 RCTs, 32 trials were retrieved for detailed evaluation, and 19 studies [10]–[28] satisfying the inclusion criteria were finally analyzed. The quality assessment of included RCTs is shown in Table 1. The baseline characteristics of enrolled studies are shown in Tables 2 and 3. Among the included studies, 17 trials described LVEF [10]–[24], [26], [27], 8 NYHA classification [15], [17], [19]–[21], [24]–[26], 6 exercise duration [12], [16]–[18], [22], [27], 3 all-cause mortality [16], [17], [19], 4 hospitalization [13], [15], [17], [23], 3 BNP [17], [20], [28] and 3 CRP [19], [22], [28]. TMZ dosage ranged from 40 to 70 mg/day and follow-up periods from 1 to 24 months.

**Table 1:** Quality assessment of included studies.

Study	Reporting of randomization	Generation of random sequence	Blinding	Withdrawal description	Jadad score
Zhao et al., 2013	Y	Unclear	Double-blind	Y	4
Fragasso et al., 2011	Y	Computer generated	Single-blind	Y	3
Cera et al., 2010	Y	Computer generated	N	Y	3
Gunes et al., 2009	Y	Unclear	Single-blind	N	1
Marazzi et al., 2009	Y	Unclear	Double-blind	Y	4
Tuunanen et al., 2008	Y	Unclear	Single-blind	Y	2
Belardinelli et al., 2008	Y	Computer generated	Double-blind	Y	5
Sisakian et al., 2007	Y	Unclear	N	Y	2
Di Napoli et al., 2007	Y	Sealed envelope	Double-blind	Y	5
Fragasso et al., 2006	Y	Computer generated	N	Y	3
Fragasso et al., 2006	Y	Computer generated	Double-blind	Y	5
Di Napoli et al., 2005	Y	Unclear	N	Y	2
El-Kady et al., 2005	Y	Unclear	Single-blind	Y	2
Vitale et al., 2004	Y	Unclear	Double-blind	Y	4
Thrainsdottir et al., 2004	Y	Unclear	Double-blind	Y	4
Rosano et al., 2003	Y	Unclear	Double-blind	Y	4
Fragasso et al., 2003	Y	Unclear	Double-blind	Y	4
Belardinelli et al., 2001	Y	Unclear	Double-blind	Y	4
Brottier et al.,1990	Y	Unclear	Double-blind	Y	4

Y = yes; N = no.

**Table 2:** Study characteristics.

Study	Patients (TMZ/Control)	TMZ (mg/day)	Follow-up duration	Inclusion criteria	Endpoints
Zhao et al., 2013	80 (40/40)	60	6 months	Diabetes, IDCM, LVEF≤40%	Left ventricular function, exercise tolerance, CRP, BNP
Fragasso et al., 2011	44 (25/19)	60	3 months	Chronic systolic HF, NYHA II–IV, LVEF <45%,	REE, LVEF, NYHA class, QOL
Cera et al., 2010	30 (17/13)	60	6 months	Chronic stable HF, NYHA I–III, LVEF <45%	LVEF, NYHA class, electrophysiological indexes
Gunes et al., 2009	87 (51/36)	60	3 months	Chronic stable HF, NYHA II–III, LVEF≤40%	Left and right ventricular functions
Marazzi et al., 2009	47 (23/24)	40	6 months	Age ≥65 years, stable ischemic heart disease, LVEF <50%	QOL, NYHA class
Tuunanen et al., 2008	19 (12/7)	70	3 months	IDCM, LVEF <47%	Echocardiographic parameters, myocardial metabolism, blood chemistry
Belardinelli et al., 2008	35 (19/16)	60	3 months	Diabetes, stable ischemic heart disease	Myocardial scintigraphy parameters, blood biochemistry
Sisakian et al., 2007	82 (42/40)	70	3 months	Stable ischemic heart disease, LVEF <40%	LVEF, exercise tolerance, NYHA class
Di Napoli et al., 2007	50 (25/25)	60	6 months	Ischemic cardiomyopathy, LVEF <35%	Exercise tolerance, LVEF, NYHA class, BNP
Fragasso et al., 2006	65 (34/31)	60	12 months	Chronic stable HF, LVEF <45%	Cardiovascular events, hospitalization, LVEF, NYHA class, QOL, BNP
Fragasso et al., 2006	12/12	60	3 months	Chronic stable HF, LVEF≤45%	Exercise tolerance, LVEF, NYHA class, cardiac PCr/ATP ratio
Di Napoli et al., 2005	61 (30/31)	60	18 months	Ischemic dilated cardiomyopathy, LVEF <40%	All-cause mortality, NYHA class, LVEF, CRP
El-Kady et al., 2005	200 (100/100)	60	24 months	Ischemic cardiomyopathy, LVEF <50%	SPECT parameters, exercise tolerance, LVEF
Vitale et al., 2004	47 (23/24)	60	6 months	Age ≥65 years, stable ischemic heart disease, LVEF <50%	Cardiovascular events, hospitalization, LVEF, NYHA class, QOL
Thrainsdottir et al., 2004	20/20	60	4 weeks	Diabetes, stable ischemic HF, NYHA II–III, LVEF≤40%	Exercise tolerance, left ventricular function
Rosano et al., 2003	32 (16/16)	60	6 months	Diabetes, stable ischemic heart disease, LVEF <50%	Left ventricular function
Fragasso et al., 2003	16/16	60	6 months	Diabetes, ischemic cardiomyopathy, LVEF≤45%	LVEF, NYHA class, exercise tolerance, blood biochemistry
Belardinelli et al., 2001	44 (22/22)	60	2 months	Ischemic cardiomyopathy	Contractile response to dobutamine, left ventricular systolic function
Brottier et al.,1990	23(10/13)	60	6 months	Severe ischemic cardiomyopathy, NYHA III–IV	Clinical status, LVEF, cardiac volume

BNP = brain natriuretic peptide; CRP = C-reactive protein; HF = heart failure; IDCM = idiopathic dilated cardiomyopathy; LVEF = left ventricular ejection fraction; NYHA = New York Heart Association; QOL = quality of life; REE = resting energy expenditure; SPECT = single photon emission CT; TMZ = trimetazidine.

**Table 3:** Patient characteristics.

Study	Patients (TMZ/Control)	Age (Mean, years) (TMZ/Control)	Male (N) (TMZ/Control)	Ischemic cause (%)	Diabetes (%)	NYHA class	LVEF (Mean, %) (TMZ/Control)
Zhao et al., 2013	40/40	59/58	32/30	0	100	II–III	34/36
Fragasso et al., 2011	25/19	70	38	66	34	II–IV	35/35
Cera et al., 2010	17/13	65/70	15/11	60	37	I–III	38/33
Gunes et al., 2009	51/36	59/57	37/21	66	29	II–III	33/31
Marazzi et al., 2009	23/24	77/78	18/22	100	NA	I–III	<50
Tuunanen et al., 2008	12/7	59/57	10/5	0	0	NA	31/38
Belardinelli et al., 2008	19/15	54/54	16/14	100	100	NA	39/40
Sisakian et al., 2007	42/40	64/66	37/33	100	NA	II–III	35/32
Di Napoli et al., 2007	25/25	64/63	15/18	100	24	II–IV	28/30
Fragasso et al., 2006	28/27	64/66	25/25	54	7	II–IV	34/36
Fragasso et al., 2006	12/12	66	11	50	NA	NA	33
Di Napoli et al., 2005	30/31	67/69	17/18	100	20	II–IV	30/31
El-Kady et al., 2005	100/100	53/53	86/78	100	34	NA	36/37
Vitale et al., 2004	23/24	77/78	18/22	100	NA	I–III	29/29
Thrainsdottir et al.,2004	10/10	67/66	9/8	100	100	II–III	33/29
Rosano et al., 2003	16/16	66/65	11/13	100	100	NA	32/33
Fragasso et al., 2003	16/16	64	16	100	100	I–III	40
Belardinelli et al., 2001	19/19	50/54	15/16	100	NA	II–III	33/33
Brottier et al.,1990	9/11	57/62	19	100	NA	III–IV	32/29

LVEF = left ventricular ejection fraction; NYHA = New York Heart Association; TMZ = trimetazidine.

### Left ventricular function

**Figure 2:**
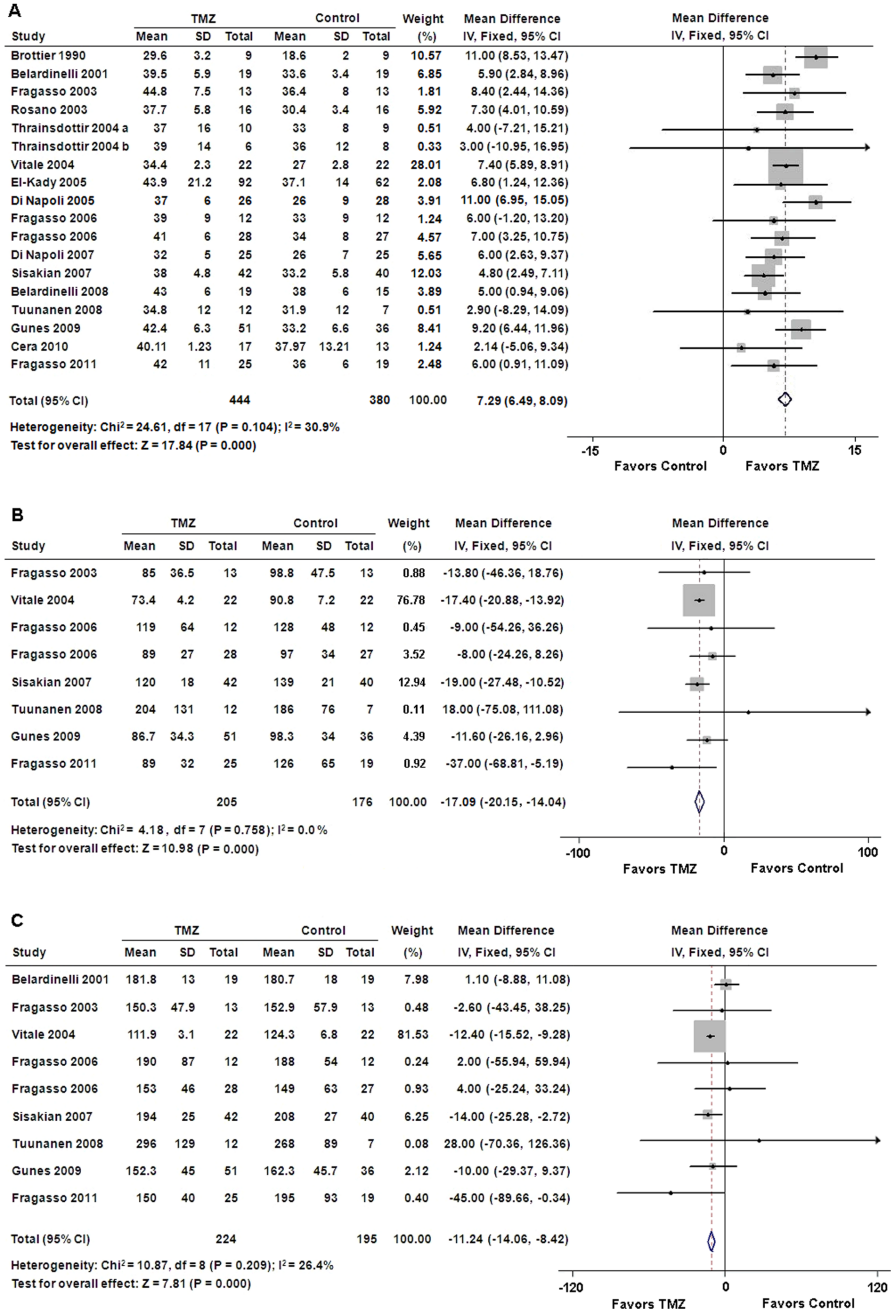
Forest plots for left ventricular function. (**A**) Left ventricular ejection fraction; (**B**) left ventricular end-systolic volume; (**C**) left ventricular end-diastolic volume. CI = confidence intervals; IV = inverse variance; TMZ = trimetazidine.

Our results indicated that additional TMZ therapy was superior to conventional treatment in terms of LVEF improvement (WMD: 7.29%, 95% CI: 6.49 to 8.09, p<0.01) (Figure 2A). In addition, LVESV and LVEDV were significantly lower in patients who received TMZ therapy than placebo treatment (WMD: −17.09 ml, 95% CI: −20.15 to −14.04, p<0.01; WMD: −11.24 ml, 95% CI: −14.06 to −8.42, p<0.01, respectively) (Figures 2B and 2C).

### NYHA classification and exercise tolerance

**Figure 3:**
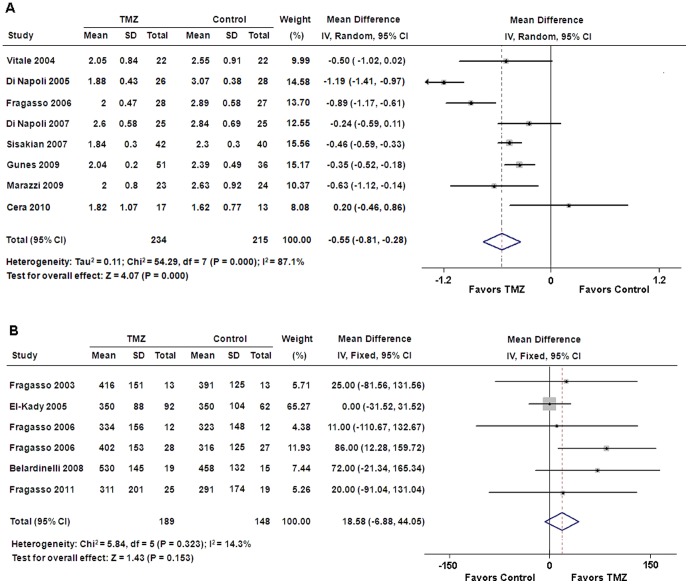
Forest plots for NYHA classification and exercise duration. (**A**) NYHA classification; (**B**) exercise duration. CI = confidence intervals; IV = inverse variance; TMZ = trimetazidine.

Pooled analysis showed that TMZ therapy resulted in a significant improvement in NYHA functional class compared with placebo control (WMD: −0.55, 95% CI: −0.81 to −0.28, p<0.01) (Figure 3A). However, there was no significant difference in exercise duration between patients treated with TMZ and placebo (WMD: 18.58 s; 95% CI: −6.88 to 44.05, p = 0.15) (Figure 3B).

### Sensitivity analysis

Since a significant heterogeneity across studies was observed for NYHA classification, we conducted a sensitivity analysis to assess the effect of each study on the pooled estimate under the random effects model. As shown in Table 4, removal of any individual study could not significantly reduce the heterogeneity.

**Table 4:** Sensitivity analysis of NYHA classification.

Study omitted	WMD (95% CI)	*P* for heterogeneity	*I^2^*
Vitale 2004	−0.55 (−0.84, −0.27)	<0.001	88.9%
Di Napoli 2005	−0.46 (−0.64, −0.27)	0.011	63.7%
Fragasso 2006	−0.49 (−0.78, −0.20)	<0.001	87.6%
Di Napoli 2007	−0.59 (−0.88, −0.30)	<0.001	88.2%
Sisakian 2007	−0.55 (−0.90, −0.21)	<0.001	88.1%
Gunes 2009	−0.58 (−0.89, −0.26)	<0.001	87.1%
Marazzi 2009	−0.54 (−0.83, −0.25)	<0.001	88.9%
Cera 2010	−0.62 (−0.88, −0.35)	<0.001	87.8%

NYHA = New York Heart Association; WMD = weighted mean difference; CI = confidence interval.

### All-cause mortality and hospitalization for cardiac causes

**Figure 4:**
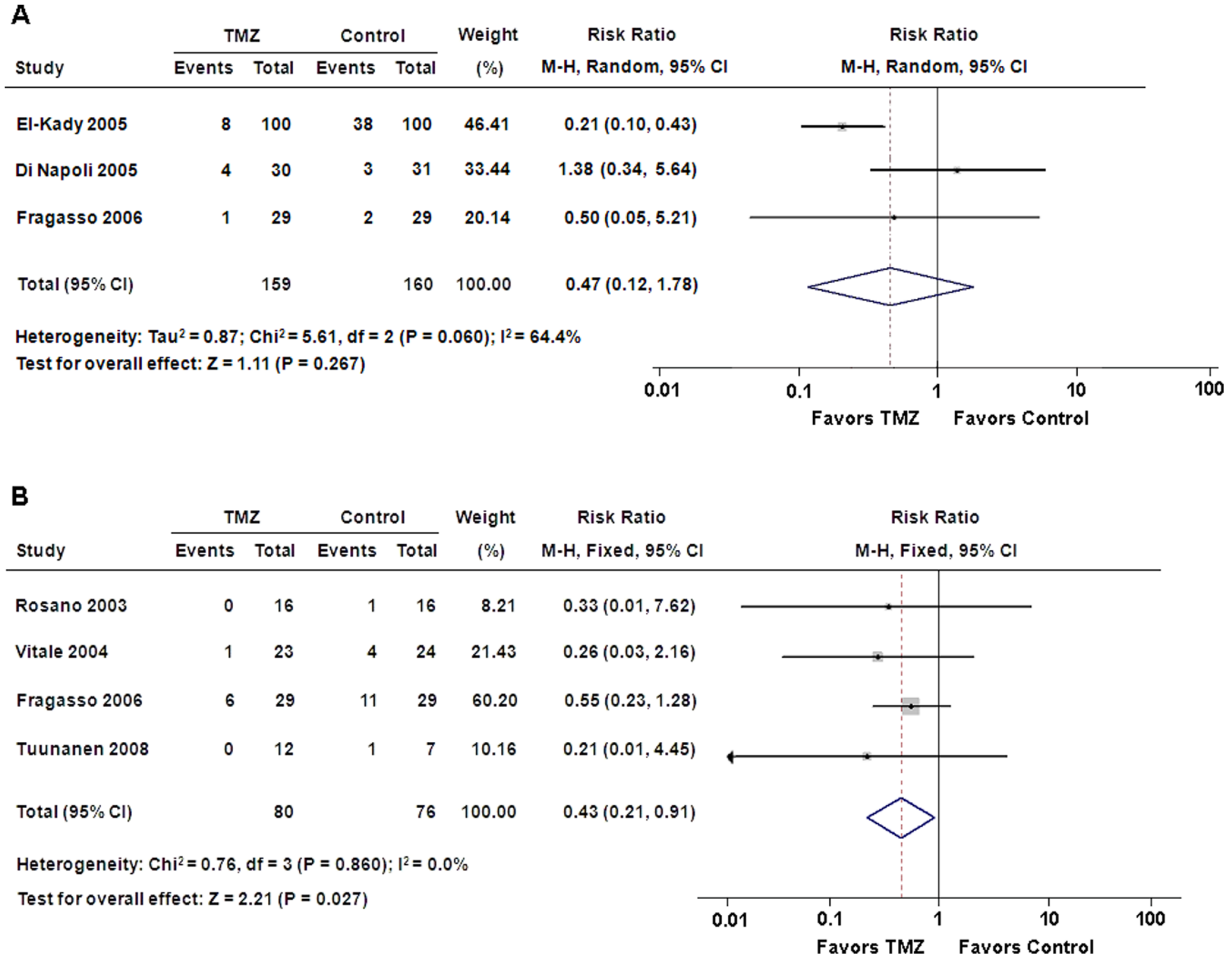
Forest plots for all-cause mortality and hospitalization for cardiac causes. (**A**) All-cause mortality; (**B**) hospitalization for cardiac causes. CI = confidence intervals; M-H = Mantel-Haenszel; TMZ = trimetazidine.

Our results suggested that there was no significant difference in all-cause mortality between patients treated with TMZ and placebo (RR: 0.47, 95% CI: 0.12 to 1.78, p = 0.27) (Figure 4A). Nevertheless, 7 of 80 patients with TMZ therapy needed hospitalization for cardiac causes, which was significantly lower than 17 of 76 patients with placebo treatment (RR: 0.43, 95% CI: 0.21 to 0.91, p = 0.03) (Figure 4B).

### Serum markers

**Figure 5:**
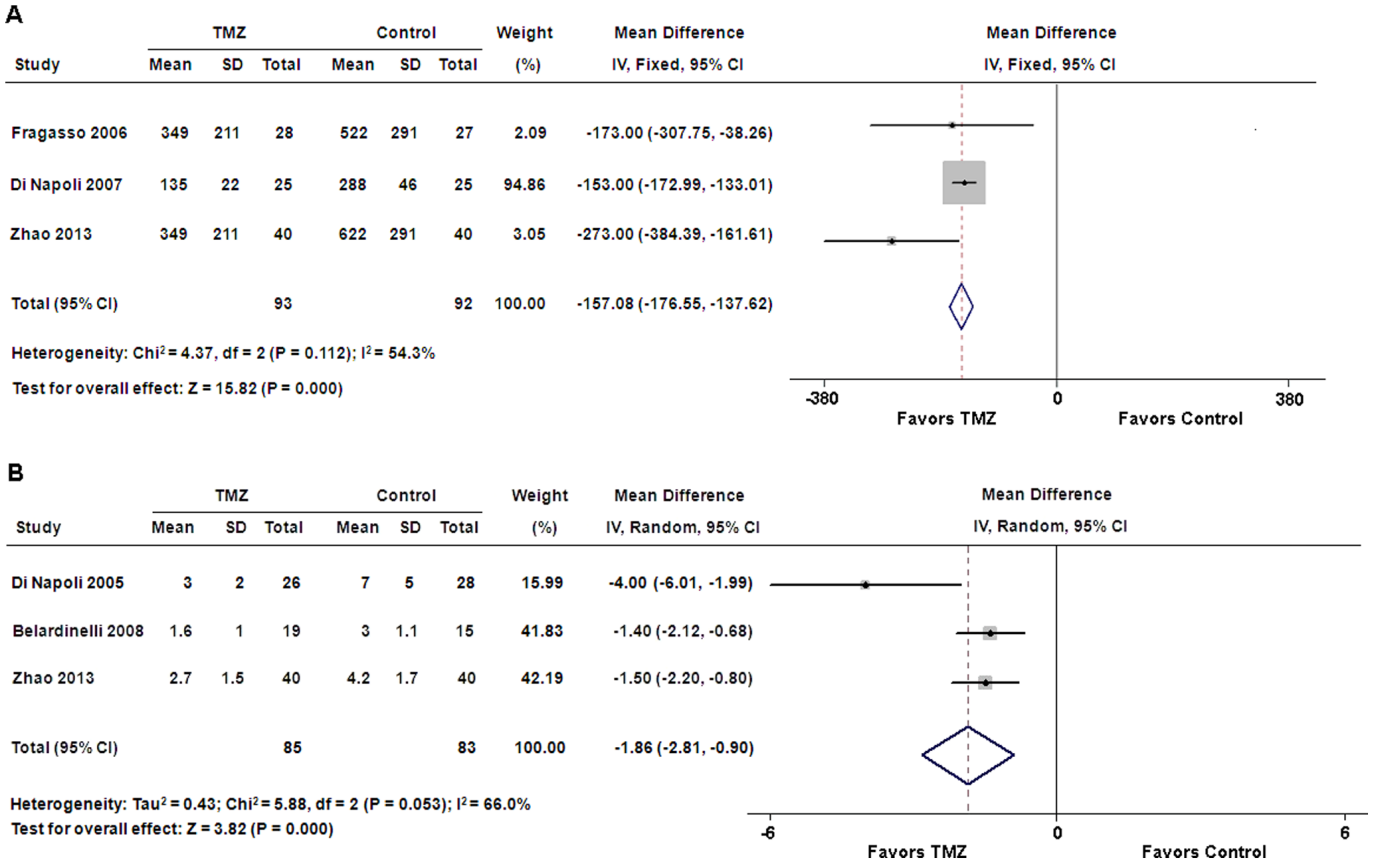
Forest plots for serum markers. (**A**) B-type natriuretic peptide; (**B**) C-reactive protein. CI = confidence intervals; IV = inverse variance; TMZ = trimetazidine.

Pooled analysis showed that serum levels of BNP and CRP were significantly decreased in the TMZ group compared with those in the control group (WMD: −157.08 pg/ml, 95% CI: −176.55 to −137.62, p<0.01; WMD: −1.86 mg/l, 95% CI: −2.81 to −0.90, p<0.01, respectively) (Figures 5A and 5B).

## Discussion

There is growing evidence that impaired carbohydrate oxidation and high rates of fatty acid oxidation contribute to the progression of myocardial dysfunction in CHF patients. TMZ has an inhibitory effect on the mitochondrial long-chain 3-ketoacyl coenzyme A thiolase, which plays a critical role in the fatty acid beta-oxidation pathway in the myocardium. As a result, there is a switch of cardiac metabolism from free fatty acid to glucose oxidation, which represents a more efficient metabolic pathway in terms of oxygen consumption and energy generation [29].

In the past few years, the beneficial effects of TMZ treatment in patients with CHF were confirmed in several small RCTs. Brottier et al. showed that TMZ improved clinical symptoms and LVEF in patients with severe ischemic cardiomyopathy [10]. Vitale et al. reported that TMZ improved left ventricular function and quality of life in elderly patients with coronary artery disease [15]. In addition, the other 4 reports of RCT revealed the protective effects of TMZ against left ventricular dysfunction in diabetic patients with ischemic cardiomyopathy [12]–[14], [22]. The cardioprotective effects of TMZ were also evaluated in patients with dilated cardiomyopathy. Tuunanen et al. reported that TMZ enhanced cardiac function and had both cardiac and extracardiac metabolic effects in idiopathic dilated cardiomyopathy with heart failure [23]. Zhao et al. indicated that TMZ treatment was associated with a considerable improvement of cardiac function and physical tolerance in diabetic patients with idiopathic dilated cardiomyopathy [28]. The pooled results of these studies suggested that TMZ therapy could significantly improve LVEF and NYHA classification in patients with CHF.

The effects of TMZ on all-cause mortality and hospitalization in CHF patients are still controversial. Only 3 reports of RCT with small samples described all-cause mortality [16], [17], [19], and 4 described hospitalization for cardiac causes [13], [15], [17], [23]. The pooled results of these studies demonstrated that TMZ treatment was associated with a significant decrease in hospitalization for cardiac causes. However, there was no significant difference in all-cause mortality between patients treated with TMZ and placebo. In this meta-analysis, we also evaluated the effects of TMZ on serum levels of BNP and CRP in patients with CHF. The pooled results suggested that BNP and CRP levels were significantly decreased in patients with TMZ treatment.

There are some differences in the pooled results between our meta-analysis and two previous meta-analyses performed by Gao et al. and Zhang et al. [7], [8]. The heterogeneities of LVEF, LVESV and LVEDV in our study were much smaller than those in the other two studies. In addition, our results showed no improvement in exercise duration and no decline in all-cause mortality in CHF patients treated with TMZ, which was not consistent with the other two meta-analyses. Furthermore, our pooled results also suggested that TMZ therapy could significantly decrease BNP and CRP levels in CHF patients.

Our study had several limitations. Firstly, the methodological quality of included studies was less than optimal, so we were not able to exclude the potential risk of bias in these trials. Secondly, the number of patients included in this meta-analysis was relatively small, so the conclusions drawn from this study should be interpreted with caution. Thirdly, the follow-up duration in these studies varied widely, from 4 weeks to 24 months.

In conclusion, our meta-analysis demonstrates that TMZ treatment in CHF patients may improve clinical symptoms and cardiac function, reduce hospitalization for cardiac causes, and decrease serum levels of BNP and CRP.

## Supporting Information

Checklist S1 **PRISMA Checklist.**
(PDF)
